# Deep learning in biology faces a transferability crisis

**DOI:** 10.1371/journal.pbio.3003656

**Published:** 2026-03-03

**Authors:** Thomas A. O’Shea-Wheller, Katie I. Murray

**Affiliations:** 1 Environment and Sustainability Institute, University of Exeter, Penryn, Cornwall, United Kingdom; 2 Centre for Ecology and Conservation, University of Exeter, Penryn, Cornwall, United Kingdom; 3 Centre for Environmental Mathematics, University of Exeter, Penryn, Cornwall, United Kingdom

## Abstract

Creating generalizable models is a conserved aim in deep learning—however, misleading claims of transferability threaten to obfuscate reliable performance evaluation. This Perspective article outlines the severity of this issue in the biosciences, and suggests potential solutions.

## Teaser

Creating generalizable models is a conserved aim in deep learning—however, misleading claims of transferability threaten to obfuscate reliable performance evaluation. We outline the severity of this issue in the biosciences, and suggest potential solutions.

The continued proliferation of artificial intelligence in both research and industry has led to a growing emphasis on the development of generalizable large-scale machine vision models. The utility of such “foundation models” lies in their ability to address broad downstream applications in areas such as species recognition [1], diagnostic imaging [2], and industrial fault detection [3]. Due to the diversity of model architectures available, substantial credence has been given to metrics that provide comparative performance evaluation, with these generally being derived from model error rates when assessing an unseen subset of test data [4]. In a research context, such metrics provide a testbed for the exploration of model generalizability, as they can quantify performance differences within and between testing domains for a given training dataset, thus assisting in the development of enhanced architectures.

However, despite their value, performance metrics and associated benchmarks are frequently used for a very different purpose—to support claims of model transferability across deployment scenarios. While superficially appealing, this relies upon a fundamentally flawed assumption, that of comparable variance distributions between testing and deployment data, and hence proportional error rates. Consequently, benchmarks face a critical limitation when used to substantiate model generalizability in any sufficiently unconstrained task. Specifically, this is that at a certain level of complexity, the test data will invariably fail to capture the full range of variation present during deployment, thus violating the central assumption of proportional error distributions.

In practice, such violations almost always occur in generalized use cases. To illustrate this, if we take the example of detecting a specific animal from camera trap data, the potential permutations of detection distance, angle, background environment, and non-target species occurrence generate a problem space dimensionality that rapidly approaches that of reality itself [5]. As such, no matter how comprehensive a testing dataset is, it is unlikely to escape context dependence, as there will always be novel sources of variability present to create further domain shifts [6]. This mismatch in construct validity is pervasive across both machine vision benchmarks and those in deep learning more broadly [7]. Indeed, standard performance metrics often not only fail to provide a meaningful measure of reliability, but may be entirely decoupled or even inversely related to true error rates [8]. It is thus not unreasonable to suggest that most, if not all, current metrics of model transferability can be seen as severely misleading unless applied within highly constrained scenarios.

The extent of this issue is exemplified by cases from ecology and medical imaging. Recent work assessing pose estimation in bird flocks has shown that the highest scoring models in terms of machine vision metrics fail to produce the most accurate results when manually ground-truthed [8]. Similarly, when exposing imaging models to adversarial examples during skin lesion classification and whole brain segmentation, analyses have found that resultant robustness is not predicted by initial accuracy or Dice overlap measurements [9]. Furthermore, even models trained on large, diverse, multi-site datasets may still fail to generalize in novel contexts. For example, assessments of a convolutional neural network trained on over 3 million camera-trap images from 18 studies across the United States found that while it achieved ~97% accuracy within its training domain, this dropped to as low as ~36% when applied to other datasets within the same region [6]. In parallel, a study comparing model error detection across six large-scale medical imaging datasets demonstrated that improved out-of-distribution performance during testing did not translate into enhanced in-domain misclassification detection [10].

How do such mismatches between metric-based evaluation and actual performance arise? At a basic level, these discrepancies occur because the error rates derived from benchmarking are not representative of those encountered during deployment. This stems from the inability of testing datasets to fully reflect the variance structure of prospective novel data, leading to poor transferability through both domain shifts and false positives [11]. More fundamentally, the continued prevalence of this issue indicates a foundational underestimation of the true depth of problem spaces in complex tasks. The resultant dilemma faced by performance benchmarks is that no matter how extensive their image repertoires may be, these will always represent a set of context-dependent, specific, and finite examples [7]. Indeed, a model trained using images from conceptual categories such as “bottle” or “phone” will have no ability to identify cases that fall outside of the specific visual distributions encountered during training, and thus it is a false equivalence to assume that benchmarks composed of many such categories can meaningfully test generalizability [12]. Perhaps most intriguingly, there appears to be an inherent assumption of conceptual understanding in machine vision models, in that when given sufficient examples of a target, they will be able to grasp the abstract concept underlying it, as is the case for humans. While this idea is readily falsifiable [13], inflated claims of benchmark generalizability paired with the widespread use of associated ranking metrics serve to continue its proliferation [7].

To address the present situation and improve the transferability of model evaluation metrics, we propose two specific actions. First, there is a need for transparency and acknowledgement of what given performance metrics truly relate to. When quoting accuracy measures derived from training data, researchers should explicitly specify and provide the testing images used to generate these values, outline the exact parameters under which they may be expected to generalize, and detail any associated caveats. This will allow potential users to compare the attributes of testing data to their own deployment scenarios, and limit the spread of misleading claims derived from context-specific benchmarks.

Second, in the absence of reliable metrics, we suggest increased adoption of an existing solution that allows users to rapidly evaluate the transferability of models utilizing their own data—namely model preview tools. Such tools enable instant application of hosted models to users’ evaluation datasets, and are commonly employed on open source repositories such as Hugging Face. By directly applying inference to ground-truthed annotations provided by the user, these utilities transfer benchmarking from a global process that must approximate all potential deployments *a priori*, to a local one that derives performance metrics specifically from the desired use case. Notably, while such tools are readily available, their apparent absence from the scientific literature suggests that there is strong potential for expanded use. As such, allowing for scalability limitations [14], enhanced requirements for authors to provide links to these tools within publications would substantially improve the ability of readers to validate model transferability claims.

While these strategies have the potential to improve the estimation of model transferability in the short term, there is a broader need for reassessment of the current model evaluation paradigm. Central to this, is the question of how and if it is possible to develop generalized benchmarks that meaningfully encapsulate transferability, reliability, and robustness in a minimally application-biased manner. Until such a time as this is answered, the only true evaluation for a specific deployment will be to conduct testing on data from that same deployment. Although here we have focused upon machine vision, this issue has far wider implications for deep learning *sensu largo*, and as such, warrants further discussion at a fundamental level.
